# Pakistan and COVID-19: The mystery of the flattened curve

**DOI:** 10.7189/jogh.11.03013

**Published:** 2021-01-11

**Authors:** Shoaib Ahmad, Don Eliseo Lucero-Prisno, Mohammad Yasir Essar, Hiba Khan, Attaullah Ahmadi

**Affiliations:** 1 Punjab Medical College, Faisalabad, Pakistan; 2 Faisalabad Medical University, Faisalabad, Pakistan; 3 Department of Global Health and Development, London School of Hygiene and Tropical Medicine, London, United Kingdom; 4 Faculty of Management and Development Studies, University of the Philippines (Open University), Los Baños, Laguna, Philippines; 5 Medical Research Center, Kateb University, Kabul, Afghanistan; 6 Dubai Medical College, Dubai, UAE; 7 Global Health Focus Asia, Kabul, Afghanistan

The novel coronavirus (COVID-19) is a public health emergency of international concern that has triggered a huge burden on the world, specifically developing countries [1]. As of 24 September in Pakistan, a total of 308 208 positive cases have been confirmed with a total of 6437 people having succumbed to the virus. Very surprisingly, Pakistan is the 128^th^ on the list of positive COVID-19 cases per a population of one million and 113^th^ in the list of COVID-19 related deaths per a population of one million [2]. This was a completely unforeseen set of results as Pakistan, a developing country, was deemed to be one of the least prepared countries to face a pandemic of this nature because of its fragile health care system, lack of resources, and low literacy rate across the country [3].

## RESPONSE OF PAKISTAN TO THE COVID-19 PANDEMIC

Lockdown, contact tracing, and case isolation are measures that have been proven to be effective in controlling an outbreak [4]. Consequently, the Pakistani government took rigorous measures to control COVID-19 spread initially by imposing a comprehensive lockdown [5]. However, the country soon gave up this pursuit and the lockdown was lifted in the first week of May, 2020, as the public was urged to live with the virus despite the rising cases. The World Health Organization (WHO) suggested on 10 June 2020 to re-impose a partial lockdown but the prime minister refused [6]. He insisted that the economy could not survive a further lockdown with more job losses and increasing numbers of hungry people.

Overall, lack of lockdown-regulations, negligible implementation of preventive measures, lack of preventive resources, lack of knowledge in the public, mass gatherings in worship places, and an already fragile health care system, had put Pakistan on a road destined to make it one of the worst hit countries by this pandemic [7]. A study by Imperial College London had predicted by various algorithms that Pakistan would report the highest number of deaths on the 10th of August 2020 with the total reaching 78 515 after which Pakistan would witness a decline [8].

**Figure:**
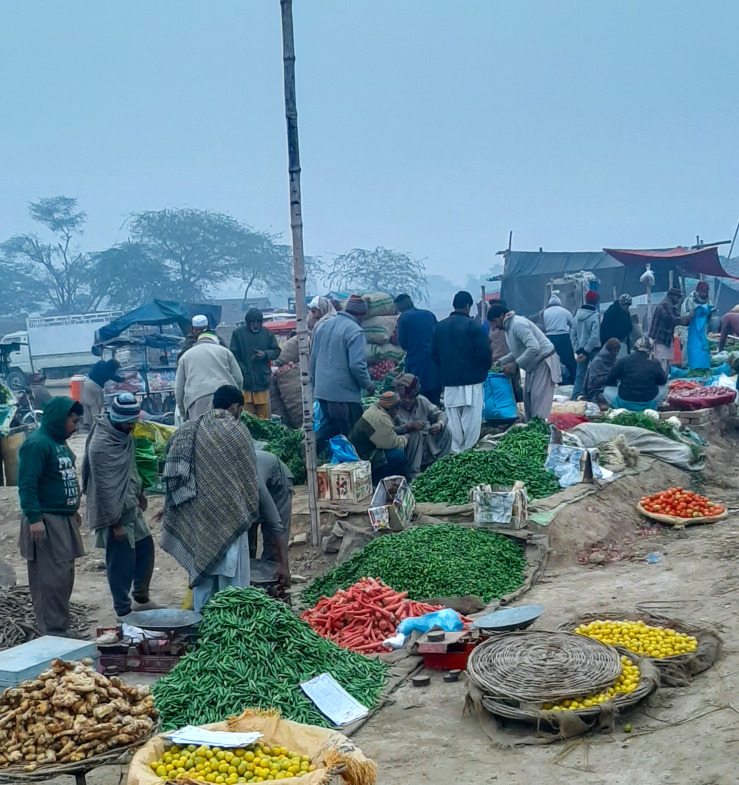
Photo: People busy in their routine and not adhering to preventive measures amidst the COVID-19 pandemic (from the author’s own collection, used with permission).

## FLATTENED CURVE

**Figure 1:**
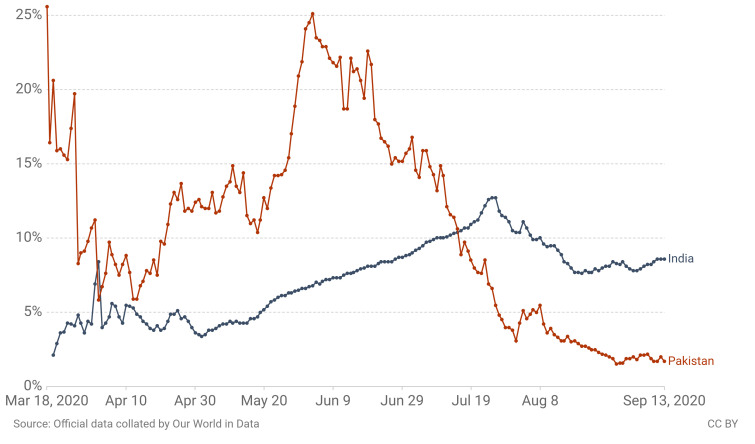
Positivity rate of COVID-19 tests for Pakistan in comparison to India [10]. The data are rolling 7-day average (the number of confirmed cases divided by the number of tests, expressed as a percentage). Tests may refer to the number of tests performed or the number of people tested, depending on which is reported by the particular country. Source: https://ourworldindata.org/coronavirus.

Views on the mystery of flattened curve in Pakistan:

- The very possible confounding factor of decreased number of cases is the disparity in reporting the data and the government trying to delude the public to prove its policies to be stronger than in reality. On the other hand, this can be contradicted by the evidence of no spike in graveyard data and the continuous fall in demand for pressurized oxygen and ventilators. The claims by very credible health care professionals of decreased hospital workload also support this theory of low intensive healthcare demand. Moreover, Pakistan managed to pay heed to other epidemics like polio and was able to carry out 4 immunization drives unlike other endemic countries like Afghanistan [9].
- Another theory to the flattened curve is the decreased number of tests conducted. However, this factor also cannot be attributed to a seemingly controlled outbreak as the positivity rates are very low, i.e. no. of confirmed cases/total no. of tests conducted [10]. The average positivity rate for Pakistan is less than 5% in August 2020 and declines further below 2% by September 2020 as shown in **Figure 1**. This figure is well below the WHO recommended figure of 5% which when achieved means the outbreak is well under control.

Nonetheless, the controlled outbreak can be due to a number of factors with the most proposed ones being:

- A non-specific immunity due to immunization by various vaccines like BCG but there are still detailed clinical trials required to develop a causal relationship [11].
- The humidity in the current monsoon season and a comparatively higher temperature which is thought to attribute to the decreased spread of virus [12].
- Pakistan’s government has had a lesser stringency index than their neighboring countries like India for example, as Pakistan preferred to impose a “smart” lockdown instead [10]. This stringency index is calculated by several policies imposed by the government including school and work-place closure, restrictions on gatherings, transport restrictions, and stay at home requirements. Since climate conditions of both countries are alike and the immunization status of both Pakistan and India are not so different, the stringency index can be the one major difference that might have turned the tide in Pakistan’s favor but there is still no evidence in corroboration of this theory.

## CONCLUSION

To conclude, it is a fact that Pakistan has gotten an optimal hold of the coronavirus outbreak considering their lack of preventative measures. However, declaring victory can be a major blunder as daily cases are still being reported in hundreds. Pakistan should be well prepared for a second wave as the government has already opened schools and universities starting from September 15, 2020. The factors adding up to this recovery are still ambiguous as neighboring countries with similar factors and behaviors are still struggling. The sequence of events and measures taken by Pakistan need detailed surveillance as it can help us flatten the curve in other countries by replicating similar measures.
